# FAS2FURIOUS: Moderate-Throughput Secreted Expression of Difficult Recombinant Proteins in *Drosophila* S2 Cells

**DOI:** 10.3389/fbioe.2022.871933

**Published:** 2022-05-05

**Authors:** Jesse A. Coker, Vittorio L. Katis, Michael Fairhead, Anja Schwenzer, Stine B. Clemmensen, Bent U. Frandsen, Willem A. de Jongh, Opher Gileadi, Nicola A. Burgess-Brown, Brian D. Marsden, Kim S. Midwood, Wyatt W. Yue

**Affiliations:** ^1^ Centre for Medicines Discovery, Nuffield Department of Medicine, University of Oxford, Oxford, United Kingdom; ^2^ Kennedy Institute of Rheumatology, University of Oxford, Oxford, United Kingdom; ^3^ ExpreS^2^ion Biotechnologies, SCION-DTU Science Park, Hørsholm, Denmark

**Keywords:** insect cells, recombinant proteins, S2 cells, glycoproteins, secreted proteins, structural biology

## Abstract

Recombinant protein expression in eukaryotic insect cells is a powerful approach for producing challenging targets. However, due to incompatibility with standard baculoviral platforms and existing low-throughput methodology, the use of the *Drosophila melanogaster* “S2” cell line lags behind more common insect cell lines such as Sf9 or High-Five™. Due to the advantages of S2 cells, particularly for secreted and secretable proteins, the lack of a simple and parallelizable S2-based platform represents a bottleneck, particularly for biochemical and biophysical laboratories. Therefore, we developed FAS2FURIOUS, a simple and rapid S2 expression pipeline built upon an existing low-throughput commercial platform. FAS2FURIOUS is comparable in effort to simple *E. coli* systems and allows users to clone and test up to 46 constructs in just 2 weeks. Given the ability of S2 cells to express challenging targets, including receptor ectodomains, secreted glycoproteins, and viral antigens, FAS2FURIOUS represents an attractive orthogonal approach for protein expression in eukaryotic cells.

## 1 Introduction

Recombinant protein expression in eukaryotic insect cell–derived systems, particularly *Spodoptera frugiperda* (Sf9/Sf21) and *Trichoplusia ni* (BTI-TN-5BI-4 or High-Five™), greatly expanded the number and complexity of proteins amenable to biochemical, structural, and functional studies. Insect cells can accommodate many posttranslational modifications and handle challenging folds, including human integral membrane proteins, and are easier, cheaper, and quicker to handle than comparable human cell–derived systems such as HEK293 or CHO. As a result, insect cell systems are routinely used for both bespoke and high-throughput protein expression, with innovative baculoviral technologies such as Bac-to-Bac^®^, *flash*BAC™, MultiBac™, and FlexiBAC allowing robust production of single proteins and multiprotein complexes (Luckow et al., 1993; Fitzgerald et al., 2006; Hitchman et al., 2010; and Lemaitre et al., 2019).

However, these baculoviral innovations failed to increase the uptake of an orthogonal insect cell line derived from *Drosophila melanogaster*: Schneider 2 cells (S2). Baculoviral systems rely on the *Autographa californica* nuclear polyhedrosis virus (AcNPV) which can only propagate in the lepidopteran species of insect cells, preventing its use in S2 and other *D. melanogaster*–derived cell lines. While some evidence suggests that promoter and protocol variations can rescue baculovirus-mediated gene delivery in *Drosophila,* nearly all commercial insect cell expression systems rely on a polyhedron promoter that is incompatible with *D. melanogaster* (Carbonell et al., 1985; Lee et al., 2000).

The *Drosophila* Expression System (DES™), marketed by Invitrogen, overcomes this challenge *via* bypassing the baculoviral stage and instead relies on directly co-transfecting a selection plasmid with a target-containing plasmid to select polyclonal stable cell lines. The DES™ expression system has been successfully used to generate many valuable proteins, but to our knowledge it has never been adapted to the high-throughput (HT) requirements of many structural biology laboratories (Moraes et al., 2012). At the Centre for Medicines Discovery (CMD), we routinely use and share protocols for HT baculoviral–mediated expression of proteins in Sf9 and High Five™ cells for structural characterization (Savitsky et al., 2010). However, even these robust pipelines failed as we pushed toward more challenging targets, particularly receptor ectodomains, secreted glycoproteins, and extracellular matrix components, which in preliminary experiments expressed well in S2 cells.

Secreted protein expression in S2 cells offers particular advantages for structural laboratories. Due to their smaller size, S2 cells grow much faster and to much higher densities (>6.0 × 10^7^ cells/ml) than other insect cells, reducing culture volumes and cost (Pamboukian et al., 2008). S2 cells tolerate diverse growth conditions, including multiple serum-free and serum-containing commercially available media; all culture styles including T-flasks, shake flasks, and bio-reactors; and a wide temperature range (22–30°C) (Moraes et al., 2012). S2 cells are capable of complex eukaryotic posttranslational modifications, including mostly paucimannosidic N-linked glycosylation (Vandenborre et al., 2011). S2 cells are “powerhouse” protein secretors, with recombinant protein yields >30 mg/L allowing industrial-scale production of challenging proteins, including a malaria vaccine, West Nile virus vaccine, HER-2 vaccine, virus-like particles, and antibodies (Adriaan de Jongh et al., 2013). S2-derived proteins led to the deposition of more than 60 structures into the PDB, including receptor ectodomains, viral glycoproteins, enzymes, hormones, and growth factors. Interested readers are directed to excellent reviews which cover the details of S2 systems (Moraes et al., 2012; Adriaan de Jongh et al., 2013; Zitzmann et al., 2017).

Although S2 cells have been used to produce many cytosolic and even membrane proteins, particularly GPCRs (Perret et al., 2003), the real strength of the system, in our experience, is for secreted and “secretable” proteins. Because of their origin from *Drosophila* macrophages, S2 cells are uniquely tuned for protein secretion and in our hands outperform the more common High Five™ cell line, in part because S2 cells grow to significantly higher cell densities. Secreted proteins undergo packaging in the endoplasmic reticulum and Golgi apparatus, where resident chaperones can help re-fold and stabilize flexible, disulfide-bonded, partially disordered, or multidomain proteins. Proteins targeted for secretion are also subject to glycosylation, which is often critical for protein stability and activity. Furthermore, purification of secreted proteins bypasses cell lysis, simplifying the procedure and protecting fragile proteins from highly active intracellular proteases. As a result, secreting recombinant proteins can increase or even rescue target expression relative to standard cytosolic strategies. At the CMD, we found this to be particularly true for complex human proteins, particularly receptor ectodomains and extracellular matrix components, which we struggled or failed to express in the cytosol.

Motivated to implement a robust and orthogonal protein secretion platform for our challenging targets, we initiated a collaboration with the Danish company ExpreS^2^ion Biotechnologies. Founded in 2010, ExpreS^2^ion Biotechnologies markets an S2-based protein secretion system called ExpreS^2^, which has been used to express over 300 recombinant proteins to date. The ExpreS^2^ platform relies on the “pExpreS^2^” class of vectors which place genes of interest downstream of a strong, constitutive, and fused Actin-Hsp70 core promoter and upstream of an opie2 polyadenylation sequence. The pExpreS^2^ vector backbones include either a Zeocin^R^ (pExpreS^2^-1) or G418^R^ (pExpreS^2^-2) marker, allowing stably expressing polyclonal cell lines to be generated without co-transfection of a selection plasmid. The ExpreS^2^ platform also includes a flexible and insect cell–optimized proprietary transfection reagent, although other commercial transfection reagents also suffice. Recent successes with the ExpreS^2^ platform include the expression and structural characterization of human endothelial protein C receptor (EPCR), production of cGMP compliant *P. falciparum* RH5 protein vaccines, and production and structural elucidation of the three-component *P. falciparum* invasion complex (Lau et al., 2015; Hjerrild et al., 2016; and Wong et al., 2019). Due to the simplicity of the one-plasmid system and the reported successes from other structural laboratories, we selected the ExpreS^2^ system as the foundation for our secreted protein pipeline.

**FIGURE 1:**
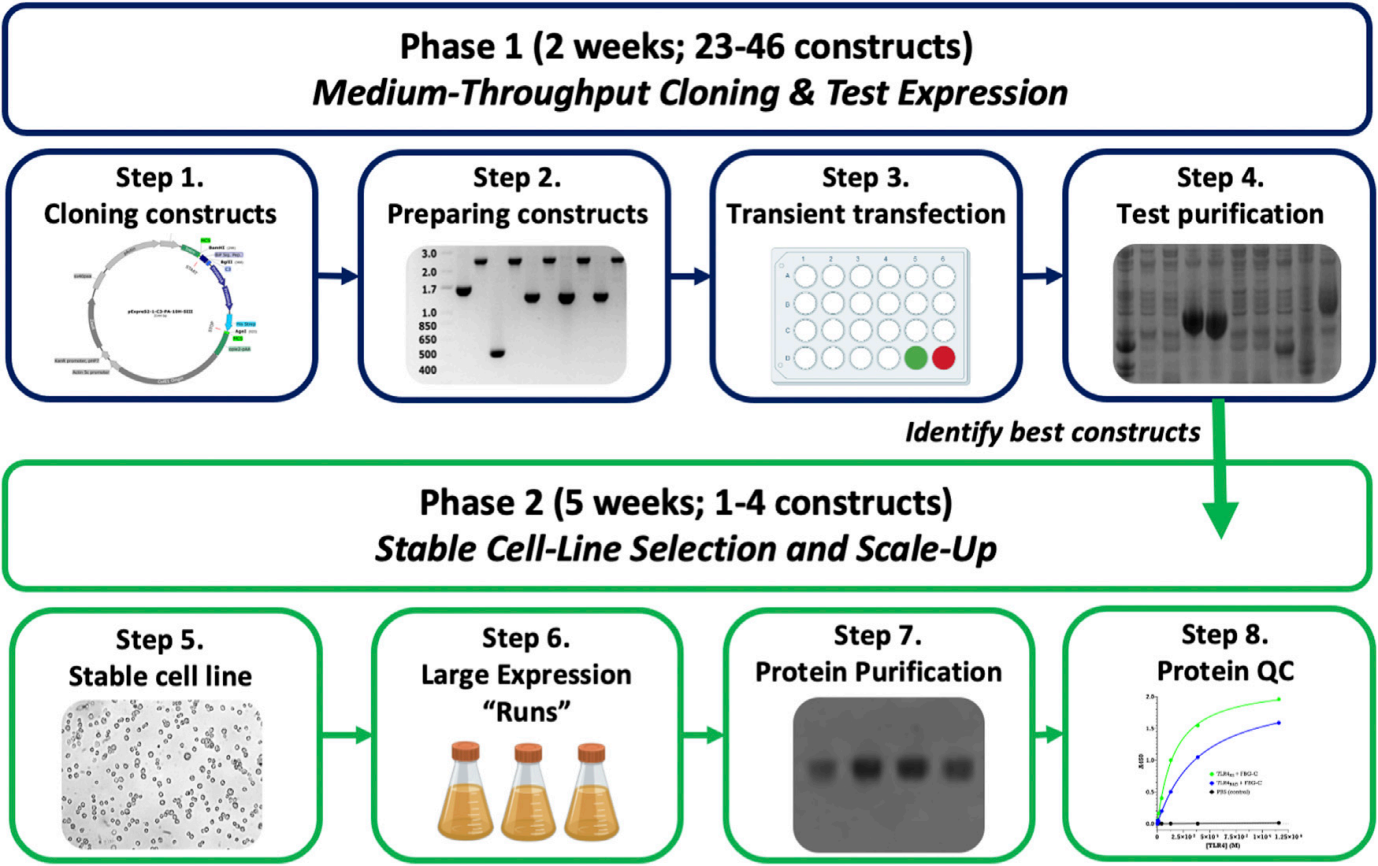
Overview of the FAS2FURIOUS method. Step 1: constructs are designed and cloned into pExpreS^2^-1 class vectors using a HiFi assembly in 96-well plates. Step 2: insert-containing clones are verified by colony PCR and mini-prepped on spin-columns. Step 3: constructs are transiently transfected in parallel into S2 cells in 24-well plates. Step 4: supernatants are harvested, test-purified using 96-well filter plates, and checked for expression on SDS-PAGE. Step 5: polyclonal stable cell lines are selected using Zeocin for constructs of interest. Step 6: polyclonal cell lines are scaled up in 1L shake flasks for large-scale purification.

Nevertheless, we needed to make a variety of improvements to the commercially available ExpreS^2^ system to facilitate a moderate-throughput pipeline for structural work. Our changes, presented and utilized herein, include the following: 1) modification of the pExpreS^2^ vectors to encode an N-terminal BiP secretion signal and C-terminal cleavable affinity epitopes including 10His, Twin-Strep, and Protein A; 2) standardization of the pExpreS^2^ vectors for high-throughput cloning; 3) quickening and miniaturizing the existing transient transfection protocol to allow parallelized expression screening; and 4) piloting the method with a variety of challenging human proteins. The resulting methodology, outlined in Figure 1, facilitates a transient test expression of up to 46 constructs in just 2 weeks without requiring bacmid or virus production, followed by stable cell line generation and large-scale purification of expressing constructs over five additional weeks. Expression testing in our pipeline is as rapid as in *E. coli* systems, and the final stable cell lines are cheaper, easier, and more reliable to scale-up for production than baculovirus-infected insect cells. The protocol presented herein, which we dubbed “FAS2FURIOUS” or “F2F,” should allow other structural laboratories to quickly deploy the gold-standard S2 secretion system to enable the characterization of previously intractable targets.

## 2 Materials and Methods

### 2.1 Protocol and Reagent Availability

A detailed step-by-step protocol for the entirety of the FAS2FURIOUS method is available in Supplementary Material. All vectors reported and used herein are available from Addgene (Plasmids #175360, #175362, #175363, #175443, #175444, #175445, #175446, and #175447).

### 2.2 Molecular Biology

#### 2.2.1 Starting Vectors

pExpreS^2^-1, pExpreS^2^-2, and pExpreS^2^-1-GFP were purchased from Expres^2^ion Biotechnologies. *Drosophila* codon–optimized TLR4, without a tag, and MD-2, cloned with a C-terminal thrombin-cleavable Protein A tag, were generously provided by Toshiyuki Shimizu from the University of Tokyo. Professor Shimizu’s TLR4/MD-2 constructs were provided in the pMT-BiP-V5-His (Invitrogen) backbone and cloned in-frame with the vector’s BiP signal peptide.

#### 2.2.2 Creation of F2F Vectors

To create the pExpreS^2^-1-CR-PA, the cassette encoding BiP-MD-2-Thrombin-Protein A was excised from pMT-BiP-V5-His *via* BamHI/AgeI restriction sites and transferred into the MCS of pExpreS^2^-1 linearized with the same restriction enzymes. This maintained the BglII cut site in between the BiP signal peptide and MD-2 open-reading frame which was present in the Shimizu entry constructs. The MD-2 insert, along with accessory restriction cloning scars from the original cassette, was removed *via* PCR and the Q5 Site-Directed Mutagenesis Kit (NEB), yielding the final pExpreS^2^-1-CR-PA. To create the pExpreS^2^-1-CR-PA-10H-SIII, the pExpreS^2^-1-MD-2-Protein-A intermediate vector was used as a template to add a 10His-SIII tag *via* one cycle of PCR mutagenesis, and then the MD-2 insert was removed with a second cycle of PCR mutagenesis as before to yield pExpreS^2^-1-CR-PA-10H-SIII. pExpreS^2^-1-C3-PA and pExpreS^2^-1-C3-PA-10H-SIII were created similarly to their thrombin-cleavable counterparts, except the thrombin cut site which was also simultaneously mutated to a C3 cut site. Finally, to generate pExpreS^2^-C3-10H-SIII, both the MD-2 insert and the Protein A tag were removed from the pExpreS^2^-1-MD-2-Protein-A-10H-SIII intermediate vector *via* PCR mutagenesis. All F2F vectors were validated by Sanger sequencing.

#### 2.2.3 Cloning Into F2F Vectors

For sub-cloning, F2F vectors were first linearized *via* BglII digestion, which cuts all F2F vectors in between the 5′ BiP signal peptide and the 3′ tags. The inserts were amplified *via* PCR, with primers containing the following HiFi Assembly overhangs:

**Table:** 

Vector	5′ Forward overhang	3′ Reverse overhang
pExpreS2-1-C3-10H-SIII	5′- cct​ttg​ttg​gcc​tct​cgc​tcg​gg	5′- ccc​tga​aac​aga​acc​tcc​aa
pExpreS2-1-C3-PA-10H-SIII		
pExpreS2-1-C3-PA		
pExpreS2-1-CR-PA		5′- gcc​gat​ccg​cgt​ggc​acc​ag
pExpreS2-1-CR-PA-10H-SII		

Linearized F2F vectors and amplicons were assembled using the NEBuiler^®^ HiFi DNA Assembly Mastermix according to the manufacturer’s recommendations, resulting in the seamless fusion of the insert with the 5′ BiP and 3′ tags. The constructs were validated using the Bioline MyTaq™ Red Colony PCR Kit with the following sequencing primers:

**Table:** 

Primer name	Sequence	Location
pExpreS2-1-for1	5′-aca​aga​cag​gtt​taa​gga​gac	Upstream synthetic intron
pExpreS2-2-for1	5′-ccg​gag​tat​aaa​tag​agg​cgc	Upstream HSP70 promoter
pExpreS2-rev1	5′-gcg​ctt​gaa​agg​agt​gtg​ta	Downstream opie pAA signal

Validated construct DNA was purified on a spin-column and heat-sterilized for 15 min at 95°C in preparation for transfection.

### 2.3 Cell Culture

#### 2.3.1 General S2 Handling

ExpreS^2^ cells (referred to as “S2” herein) were purchased from ExpreS^2^ion Biotechnologies. The S2 cells were regularly maintained in EX-CELL^®^ 420 (Sigma Aldrich) + 1X penicillin/streptomycin. The S2 cells were routinely split every 3–4 days to a density of 8 × 10^6^ cells/ml in round-bottomed flasks and grown at 25°C and 110 RPM.

#### 2.3.2 Transient Test Expression

The S2 cells were split to 8 × 10^6^ cells/ml by centrifugation (400 x *g* for 3 min), and 3 ml of the cells was seeded into deep-well 24-well blocks. The cells were transfected with 37.5 µl of ExpreS^2^ Insect-TRx5 (TRx5; ExpreS^2^ion Biotechnologies) and 7.5 µg of an insert-containing F2F construct. The transfected cells were allowed to grow at 500 RPM for 68–72 h at 25°C. The supernatants were harvested *via* centrifugation (1,500 x *g* for 20 min) and purified using 100 µl bed volume of the Ni-NTA resin and a 96-well deep-well filter block (ThermoFisher). The proteins were eluted in 100 µl of 1X PBS +500 mM imidazole and analyzed by SDS-PAGE. Putative target bands were confirmed using the in-gel tryptic digest LC-MS/MS.

#### 2.3.3 Stable Cell Line Selection

To select stable cell lines of well-expressing constructs, the S2 cells were split to 2 × 10^6^ cells/ml by centrifugation (400 x *g* for 3 min) and 5 ml of the cells was seeded into T25 flasks. The cells were transfected with 50 µl of TRx5 and 12.5 µg of construct DNA and rested at 25°C for 4 h before the addition of 1 ml FBS. For a positive control, one flask was transfected with pExpreS^2^-1-GFP, which can be used to track transfection efficiency (often >50%). For a negative control, one flask was transfected with TE. After 24 h, a selection reagent was added. For pExpreS^2^-1 class vectors, 2 mg/ml Zeocin was used. For pExpreS^2^-2 class vectors, 4 mg/ml G418 was used. For co-selection, 1.5 mg/ml Zeocin +4 mg/ml G418 was used. The cells were selected for 24 total days, during which every 3–4 days, the cells were split back to 1 × 10^6^ cells/ml in EX-CELL^®^ 420 + 10% FBS + selection reagent. After 24 days, all cells in the positive control were GFP-positive and all cells in the negative control were dead. At the end of the selection, the cells were transferred in 15 ml to a T75 flask with 10% FBS (without a selection marker) and then scaled up again to 25 ml in a 125-ml shake flask without FBS. Stable cell lines were then scaled-up, passaged, and frozen down in 10% DMSO as recommended by ExpreS^2^ion.

### 2.4 Purification of Recombinant Proteins

#### 2.4.1 Commercially Available Proteins

The recombinant human CD44 ectodomain derived from mammalian cells was purchased from Abcam (ab173996). Recombinant human TLR4 ectodomain, derived from mammalian cells, was purchased from R&D systems.

#### 2.4.2 TLR4 and MD-2 From S2 Cells

*Drosophila* codon–optimized human TLR4 ectodomain (UniProtKB O00206, P23-M629) and MD-2 (UniProtKB Q9Y6Y9, Q19-N160) received from Toshiyuki Shimizu (University of Tokyo) were cloned into pExpreS^2^-1-CRPA and stably expressed in S2 cells. Every 3–4 days, 0.5–1 L of the supernatant from stable cell lines was harvested and purified on a 5 ml column of IgG Sepharose 6 Fast Flow Affinity Resin (GE). The protein was eluted in 0.5M acetic acid pH = 3.4 and then immediately neutralized by desalting into 1X PBS. TLR4 was incubated with 10 U/mg human recombinant thrombin protease (EMD Millipore) for 4 h at room temperature, while MD-2 was routinely left tagged for stability. TLR4 was further purified *via* size-exclusion chromatography on a Superdex 200 16/60 column into 1X PBS, while MD-2 was polished into 25 mM Bicine pH = 8.5 + 250 mM NaCl. The proteins were concentrated to ∼5 mg/ml and frozen at −80°C for downstream applications. Protein identity was validated by intact mass spectrometry for MD-2 and tryptic digest LC-MS/MS for TLR4.

#### 2.4.3 FBG-C From *E. coli*

The C-terminal fibrinogen-like globe domain of human tenascin C (FBG-C; UniProtKB P24821 Isoform 1, I1974-A2201) was cloned using ligation-independent cloning into pNIC-NHStIIT, which places FBG-C downstream of a TEV-cleavable tandem 6His + Twin-Strep-tag^®^. FBG-C was expressed in BL21 (DE3) Tuner + CyDisCo pMJS226 *E. coli* cell lines. pMJS226, the plasmid encoding the CyDisCo components, was supplied from Prof. Lloyd Ruddock and allows the soluble expression of disulfide-containing proteins such as FBG-C (Gaciarz et al., 2017). The protein was expressed overnight at 18°C in “TB-PLUS” media (1X Terrific Broth +15 ml/L glycerol +0.01% Antifoam +1 mM MgSO_4_ + 10 mM (NH_4_)_2_SO_4_ + 0.5% (w/v) glucose + 1X *E. coli* trace metals). The cells were broken by sonication, and FBG-C was purified using StrepTactinXT™ affinity resin and eluted with 50 mM HEPES pH = 7.5, 250 mM NaCl, 5% glycerol, and 50 mM D-biotin. FBG-C was cleaved with TEV for 4 h at room temperature before a final purification step was performed on a Superdex 75 16/60 column into 20 mM HEPES pH = 7.5, 250 mM NaCl, 2% glycerol. FBG-C was concentrated to ∼20 mg/ml and flash-frozen in liquid nitrogen before storage at −80°C for downstream applications. The presence of two disulfide bonds and identity of the protein was confirmed by intact mass spectrometry.

#### 2.4.4 CD44-v10 From S2 Cells

The ectodomain of the human CD44 v10 isoform (UniProtKB P16070 Isoform 11, A20-P335) was cloned into pExpreS2-C3-10H-SIII alongside a non-cleavable, C-terminal 6His tag and a stop codon before the tags supplied in the vector. A polyclonal stable cell line was generated as before and was grown for 4 days before harvesting 50 ml of the supernatant. Imidazole was added to the supernatant (10 mM) prior to the addition of 0.5 ml of equilibrated Ni–NTA resin. Binding to the resin occurred on a rotating wheel for 1 h at 8°C, before batch-washing beads twice with 100 ml of 50 mM HEPES pH = 7.5, 500 NaCl, and 10 mM imidazole. The resin was loaded onto a poly-prep column and further washed with 5 ml 50 mM HEPES pH = 7.5, 500 NaCl and 25 mM imidazole. CD44-v10 was eluted with 2 ml of 50 mM HEPES pH = 7.5, 500 NaCl, and 250 mM imidazole. CD44-v10 was further purified on a Superdex 200 16/60 column into 20 mM HEPES pH = 7.5 and 200 mM NaCl before being concentrated to 1.5 mg/ml using a centrifugal filtration unit and stored at 80°C. The protein identity was validated by tryptic digest LC-MS/MS.

#### 2.4.5 CD44-Link From *E. coli*

The ectodomain of human CD44 containing only the Link domain (UniProtKB P16070 Isoform 1, A20-N172) was cloned into pNIC28-Bsa4, which places CD44-Link downstream of a TEV-cleavable 6His tag. CD44-Link was expressed as inclusion bodies in 1 L of Terrific Broth from BL21 (DE3) Rosetta cells. After harvesting the cells, the inclusion bodies were re-suspended into 20 ml of 20 mM Tris pH = 8.0. Washing of the inclusion bodies (up to four times) was performed by sonication (10 min), centrifugation (9,000 x *g*, 20 min, 4°C), and re-suspension into 30 ml of 50 mM Tris pH = 8.0, 100 mM NaCl, and 0.5% Triton X-100. A final wash in 50 mM Tris pH = 8.0 and 100 mM NaCl was performed to remove Triton X-100. The protein was solubilized in 10 ml of 8 M urea, 50 mM MES pH = 6.5, 0.1 mM EDTA, and 0.1 mM DTT at 4°C overnight on a rotating wheel. The protein was re-folded over 2 days at 4°C by rapid dilution into 250 mM L-arginine, 100 mM Tris–HCl pH = 8.5, 2.5 mM reduced glutathione, 2.5 mM oxidized glutathione, and 1:200 dilution of Pierce Complete EDTA-free protease inhibitors to give a final protein concentration of 50 µg/ml (typically using 1–2 L of buffer). The re-folded protein was passed through a 0.2-µm filter before being concentrated using a Vivaflow 200 (Sartorius) tangential flow filtration unit (to 50 ml), followed by a centrifugal filtration unit. Further purification and removal of aggregates were performed on a Superdex 75 16/60 column into 20 mM Tris–HCl pH = 8.0 and 150 mM NaCl. Peak fractions were concentrated to 7 mg/ml prior to storing at −80°C. The protein identity was validated by using intact mass spectrometry, revealing the correct formation of the three disulfide bonds within the CD44-Link.

### 2.5 *In Vitro* Assays

#### 2.5.1 Analytical Size Exclusion Chromatography

The TLR4:MD-2 complex was analyzed using a Superdex 200 Increase 30/100 on a Dionex Ultimate 3000 UHPLC. A280 traces reported from 100-µl injections containing 200 µg of TLR4, 400 µg of MD-2, or 200 µg of TLR4 + 400 µg MD-2.

#### 2.5.2 TLR4-Binding ELISA

The TLR4-binding ELISA was implemented essentially as previously reported (Zuliani-Alvarez et al., 2017). Briefly, 1 µg/ml of FBG-C was coated overnight onto microlon medium binding plates (Greiner Bio One 655080) before blocking for 2 h with 10% BSA in PBST. A concentration series of TLR4, dissolved in PBST +2% BSA, was prepared for both in-house–purified *Drosophila-*derived TLR4 and commercially available TLR4 from mammalian cells (R&D). After incubating for 2 h at 37°C, the plate was probed for TLR4 binding using a primary antibody (Invivogen, mAbg-hTLR4) in PBST +2% BSA for 2 h at RT. After thorough washing, the anti-rabbit HRP-coupled secondary antibody (BioRad STAR13B) was used for 1 h at room temperature before colorimetric readout using TMB. OD450 was measured using a FluoStar Omega plate reader.

#### 2.5.3 CD44-Binding ELISA

Detection of hyaluronic acid (HA) binding to CD44 was performed using a modified ELISA assay, essentially as previously described (Banerji et al., 1998). Briefly, the CD44 protein was diluted into 15 mM sodium carbonate buffer (pH 9.3), and 100 μl was added to a well of a 96-well Nunc-MaxiSorp flat-bottomed plate. Adsorption of the protein to the well was performed for 6 h at room temperature. Further adsorption was blocked by the incubation of the wells with 1% BSA in PBS and 0.05% Tween-20 at 4°C overnight. The wells were washed with PBS containing 0.05% Tween-20 prior to incubation with 100 μl of 10-kDa HA (Creative PEGWorks; HA-6011) diluted to 5 μg/ml for 1 h at room temperature. After washing, the wells were incubated for 30 min with 100 μl of streptavidin-conjugated HRP at 100 ng/ml. After a further wash, 100 μl o-phenylenediamine substrate (0.4 mg/ml in 0.4 mg/ml urea hydrogen peroxide, 0.05 M phosphate-citrate pH = 5.0) was added to the wells for 20 min. HRP activity was terminated by the addition of 50 μl 2.5 M sulfuric acid prior to the measuring activity at 492 nm.

## 3 Results

### 3.1 Modification of the pExpreS2-1 Vector for Structural Biology

**FIGURE 2:**
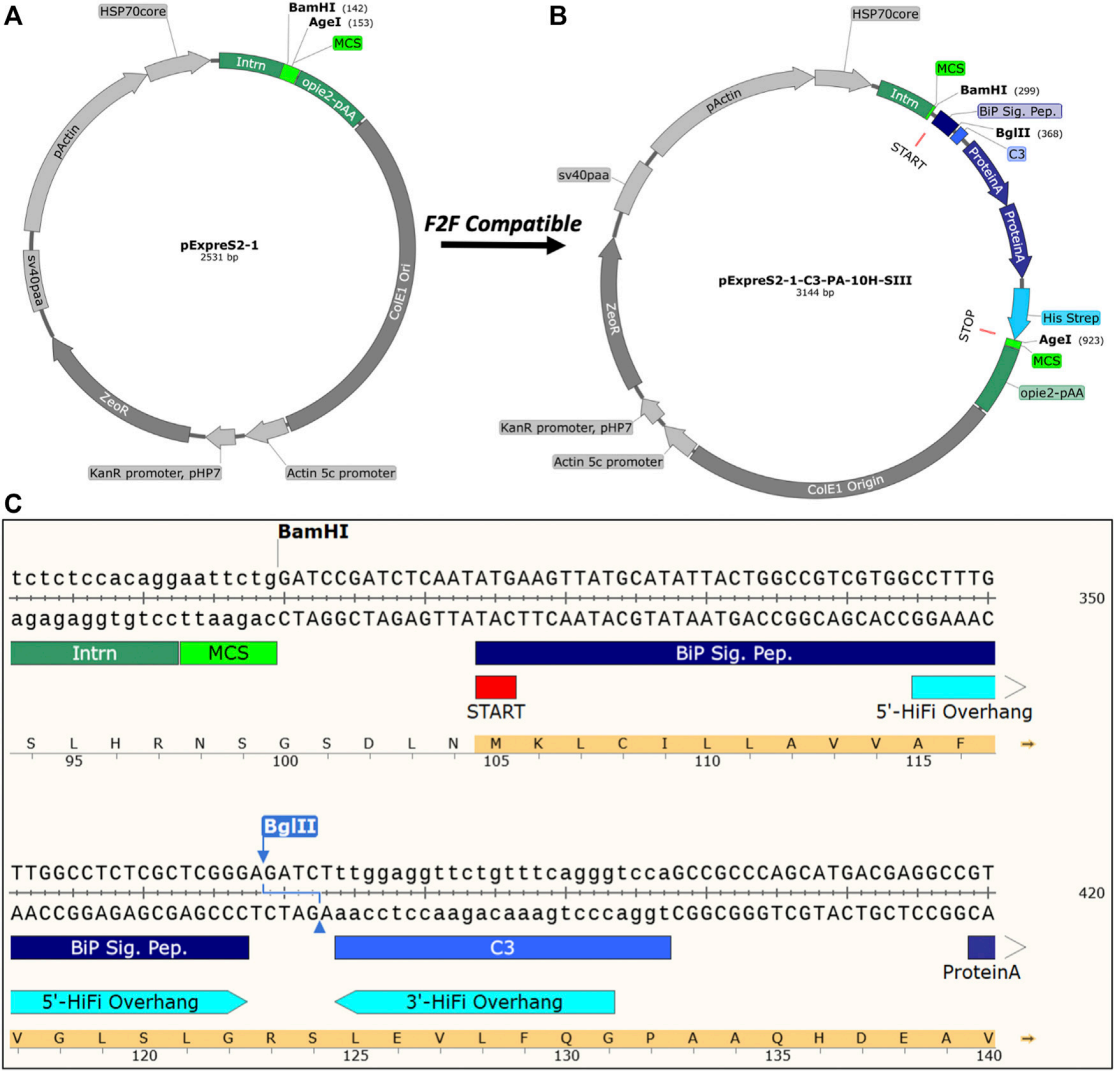
Vectors and construct design. **(A)** pExpreS^2^-1 vector backbone included in the ExpreS^2^ion’s commercially available kit. **(B)** Exemplar F2F vector, pExpreS^2^-1-C3-PA-10H-SIII, modified for high-throughput structural applications. **(C)** Sequence-level details of pExpreS^2^-1-C3-PA-10H-SIII showing the *BglII* linearization sequence, recommended HiFi overhangs, N-terminal secretion peptide, and C-terminal affinity tags. An overview of all F2F vectors created for this study can be found in Table 1.

**FIGURE 3:**
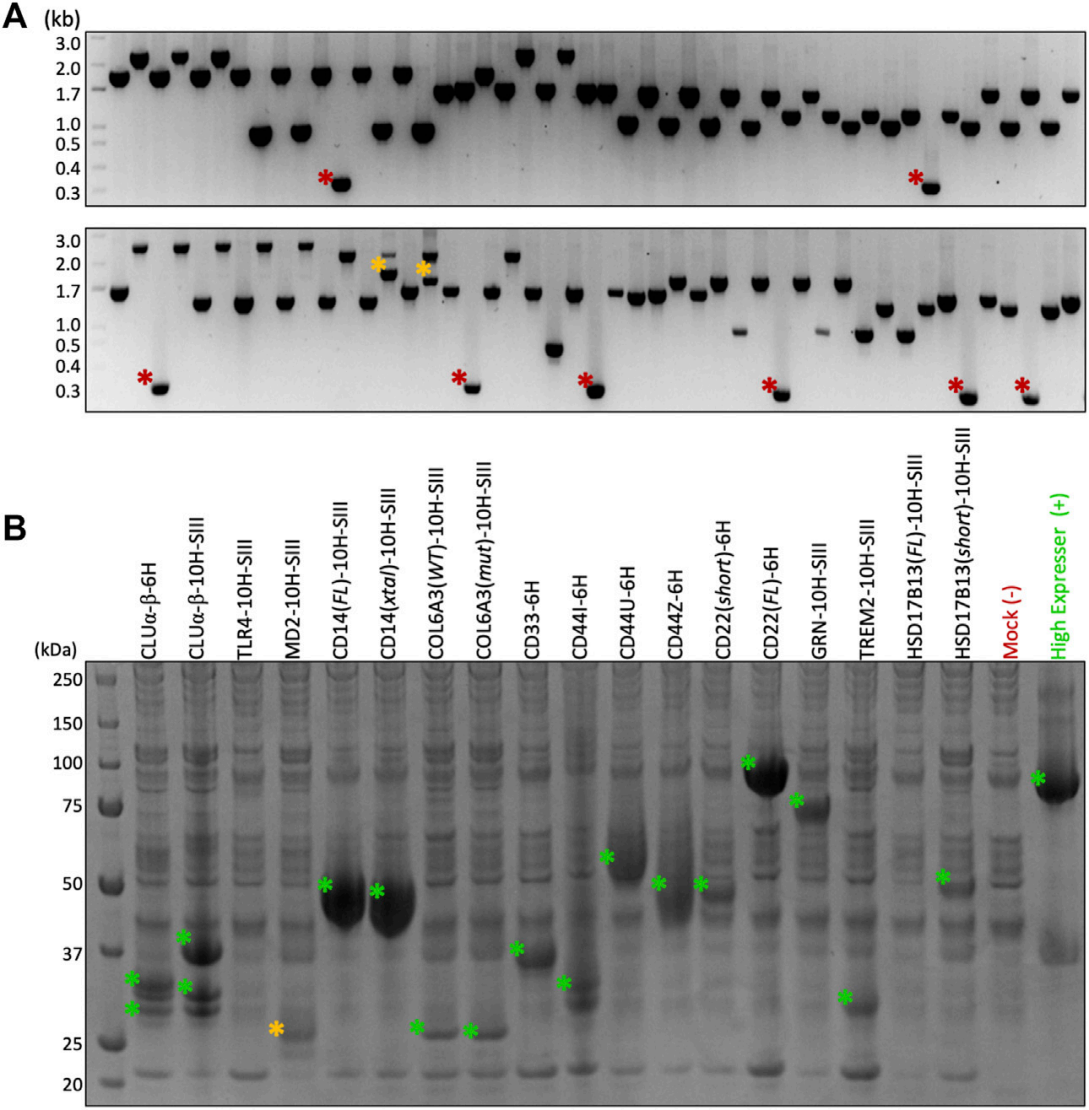
Cloning and test expression results. **(A)** Colony PCR after selecting 94 colonies from 18 different constructs demonstrates >90% assembly efficiency using HiFi. Red asterisk = vector alone; orange asterisk = unidentified products. **(B)** SDS-PAGE after 3-ml test expression and Ni-NTA pull-down. Eighteen constructs and two controls tested in parallel. Green asterisk = target band confirmed by in-gel tryptic digest MSMS; orange asterisk = putative target band.

The original pExpreS^2^-1 backbone (Figure 2A) contains only a restriction endonuclease–based (RE) multiple cloning site (MCS), which is unsuitable for high-throughput cloning due to the off-target cleavage of DNA templates (Festa et al., 2013). Furthermore, because of decreased secretion efficiencies observed for constructs with “scars” between the signal peptide and gene of interest (data not shown), we preferred a seamless cloning strategy. Therefore, we made our F2F vectors compatible with the NEBuilder^®^ HiFi DNA Assembly (HiFi), which builds upon standard Gibson Assembly methodologies and allows a seamless, one-step, and high-efficiency assembly of DNA fragments (Gibson et al., 2009). HiFi assemblies are also compatible with un-purified PCR reaction mixtures, allowing us to bypass tedious PCR clean-up steps and assemble the constructs in only 1 day. The main drawback of HiFi assemblies is the cost, which we partially mitigate by halving the recommended reaction volume without compromising assembly efficiency (Figure 3A).

**FIGURE 4:**
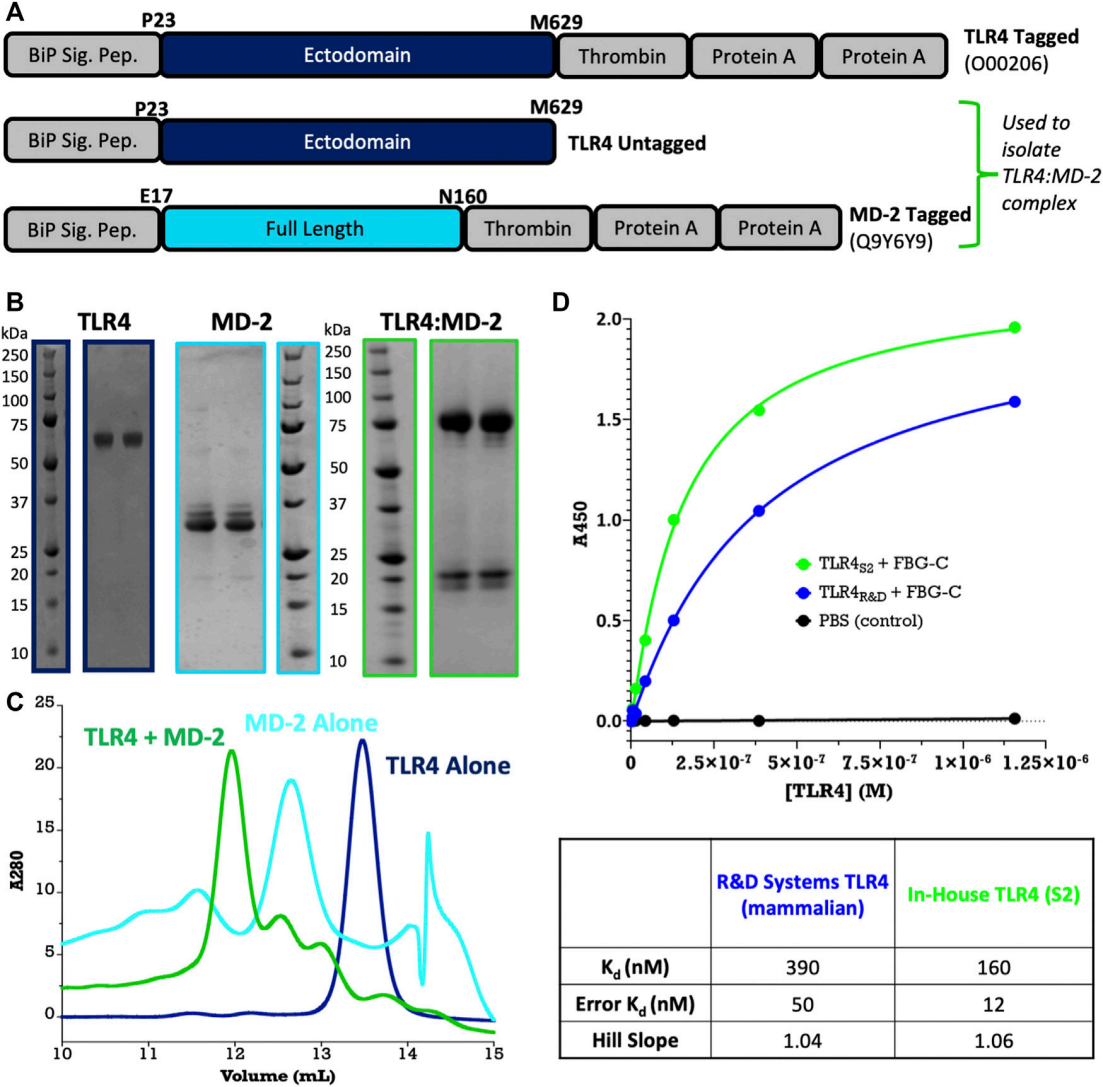
Large-scale purification of TLR4 and MD-2. **(A)** Construct design using pExpreS^2^-1-CRPA and pExpreS^2^-2. **(B)** Final purity for TLR4, MD-2, and TLR4:MD-2 complex after IgG pull-down and size-exclusion chromatography (SEC). In the TLR4 and TLR4:MD-2 samples, the Protein A tag has been removed, while in the MD-2 sample, the tag is retained for stability. All SDS-PAGE bands were confirmed by in-gel tryptic digest MSMS. **(C)** Analytical SEC binding assay shows that individually purified TLR4 and MD-2 associate into a complex as expected. It is to be noted that MD-2 exists as a mixed homo-oligomer by itself in solution and, therefore, elutes before the larger TLR4 monomer. **(D)** Activity assay confirms that TLR4:FBG-C binding is similar between S2-derived and commercially available mammalian cell-derived TLR4 from R&D Systems. Data are presented from a single biological replicate and were fit *via* nonlinear regression to a one-to-one saturation binding model using GraphPad Prism.

After selecting the overall cloning strategy, we then functionalized the pExpreS^2^-1 backbone with the requisite purification epitopes. All of our F2F vectors rely on the *Drosophila* immunoglobulin heavy chain–binding protein (BiP) signal sequence, which is commonly used for efficient protein secretion in *Drosophila* (Munro and Pelham, 1986; Sonoda et al., 2012; and Ohmuro-Matsuyama and Yamaji, 2018). At the C-terminus, we added a selection of cleavable tandem-affinity purification (TAP) tags, which enhance purification flexibility, improve final protein purity, and diversify downstream biochemical applications (Li, 2011). Alongside a standard 10His tag, which itself is sufficient for small-scale expression testing using immobilized metal-affinity chromatography (IMAC; Figure 4B), we also included two high-affinity tags designed for pull downs from dilute cell culture media samples: Protein A and Twin-Strep-tag^®^. Because of its relatively large size, the Protein A tag, derived from *Staphylococcal* protein A, can increase solubility and proteolytic stability of fusion partners; furthermore, with a K_d_ of just 10 nM for IgG, even dilute cell culture supernatants can be affinity-purified on IgG Sepharose (Kimple et al., 2013). The Twin-Strep-tag^®^, which relies on an engineered streptavidin-binding peptide, is specifically designed for the purification of recombinant proteins from dilute supernatants using the *Strep-*Tactin^®^ affinity resin (Schmidt et al., 2013). Elution from the *Strep-*Tactin^®^ affinity resin relies on excess free biotin and, therefore, is much gentler than elution from IgG Sepharose, which requires low pH. Because of these benefits, the TAP “His-Twin-Strep” tag is the workhorse affinity epitope at the CMD, and it routinely performs well across soluble, secreted, and membrane proteins.

In between the regions coding for the N-terminal BiP signal peptide and the C-terminal affinity tag(s), we placed a *BglII* cut site that is used to linearize the vector during cloning and is removed during the HiFi assembly step when our recommended overhangs are used. In addition, we included either a thrombin or human rhinovirus 3C (HRV 3C) protease site, which is used to cleave the purification tags from the final recombinant protein. HRV 3C protease, also marketed as PreScission™ protease, outperforms the more commonly used tobacco etch virus (TEV) protease across a variety of conditions, including in high-imidazole IMAC elution buffers and at 4°C (Ullah et al., 2016). Thrombin protease has a higher specific activity than HRV C3 protease; however, it sometimes suffers from off-target cleavage activity and is not available in a tagged form for easy removal (Waugh, 2011). Figure 2B shows a representative F2F vector, pExpreS^2^-1-C3-PA-10H-SIII, and Figure 2C details the architecture of the HiFi cloning region. Table 1 provides an overview of all the vectors used herein.

**TABLE 1:** Overview of all the vectors used in this study and publicly available at Addgene.

Vector name	Vector description	*E. coli* resistance	Selection marker	Cloning strategy	N-Terminal Tag	C-terminal Tag	Protease cleavage site
**pExpreS** ^**2**^ **-1** (Addgene #175360)	Parent vector from ExpreS2ion	25–50 µg/ml Zeocin	2 mg/ml Zeocin	Restriction enzyme	None	None	None
**pExpreS** ^**2**^ **-2** (Addgene #175362)	Parent vector from ExpreS2ion	100 µg/ml Kanamycin	4 mg/ml G418	Restriction enzyme	None	None	None
**pExpreS** ^**2**^ **-1-GFP** (Addgene #175363)	GFP (+) transfection control	25–50 µg/ml Zeocin	2 mg/ml Zeocin	N/A	N/A	N/A	None
**pExpreS** ^**2**^ **-1-C3-10H-SIII** (Addgene #175444)	F2F expression vector	25–50 µg/ml Zeocin	2 mg/ml Zeocin	HiFi	BiP Signal Peptide	10His + Twin-Strep	HRV C3 Protease
**pExpreS** ^**2**^ **-1-C3-PA-10H-SIII** (Addgene #175443)	F2F expression vector	25–50 µg/ml Zeocin	2 mg/ml Zeocin	HiFi	BiP Signal Peptide	10His + Twin-Strep + Protein A	HRV C3 Protease
**pExpreS** ^**2**^ **-1-C3-PA** (Addgene #175446)	F2F expression vector	25–50 µg/ml Zeocin	2 mg/ml Zeocin	HiFi	BiP Signal Peptide	Protein A	HRV C3 Protease
**pExpreS** ^**2**^ **-1-CR-PA** (Addgene #175445)	F2F expression vector	25–50 µg/ml Zeocin	2 mg/ml Zeocin	HiFi	BiP Signal Peptide	Protein A	Thrombin
**pExpreS2-1-CR-PA-10H-SIII** (Addgene #175447)	F2F expression vector	25–50 µg/ml Zeocin	2 mg/ml Zeocin	HiFi	BiP Signal Peptide	10His + Twin-Strep + Protein A	Thrombin

### 3.2 Parallelization of Transient Test Expressions

Using the new F2F vector backbones, initial experiments achieved excellent expression of a handful of difficult targets using the recommended commercial methodology for transient transfections. However, in order to fully deploy ExpreS^2^ as a pipeline secretion system for moderate-throughput structural applications, we needed to miniaturize and parallelize the reported protocols. ExpreS^2^ion Biotechnologies recommends either 1) an 8 ml transient transfection in 50-ml conical tubes using 20 µg of DNA at >0.6 µg/µl or 2) a 10 ml transient transfection in 100-ml flat-bottomed flasks using 25 µg of DNA at >0.6 µg/µl. These protocols require midi- or maxi-prepping constructs, and parallelizing transfections in falcon tubes or flasks is cumbersome. Using the established methodology, we could reasonably test just 3–4 constructs in a week, preventing us from deploying the system at the requisite scale.

Therefore, we optimized and parallelized the transfection methodology by implementing three key improvements. First, we reduced the cell volume to just 3 ml per construct, allowing us to perform transient test expressions in 24-well deep-well blocks. Second, we reduced the amount and concentration of DNA required to 7.5 µg at >200 ng/µl, which we routinely achieved with a standard miniprep of the F2F vectors. Finally, we implemented a 96-well filter-plate–based test purification protocol, which supports all HT work at the CMD and allows rapid analysis of the expression levels. Coupled with the HT-cloning methodology outlined previously, the F2F method allows either 22 (one mini-prep cycle and one test expression plate) or 46 (two mini-prep cycles and two test expression plates) constructs to be tested in parallel with the appropriate controls. The cloning and test expression stage of F2F takes only 2 weeks and does not require bacmid preparation or virus amplification; therefore, F2F is most similar to *E. coli* expression protocols and is by far the fastest eukaryotic pipeline deployed at the CMD.

### 3.3 Pilot of the Entire F2F Method

To validate the F2F method and demonstrate its robustness, we cloned and test-expressed 18 constructs containing 11 challenging gene targets, many of which expressed poorly using other systems. These “pilot” genes included receptor ectodomains such as toll-like receptor 4 (TLR4), monocyte differentiation antigen CD14 (CD14), B-cell receptor CD22 (CD22), myeloid surface antigen CD33 (CD33), CD44 antigen (CD44), and triggering receptor expressed on myeloid cells 2 (TREM2); naturally secreted proteins such as lymphocyte antigen 96 (MD-2), collagen alpha-3(VI) (COL6A3), clusterin (CLU), and progranulin (GRN); and the cytosolic protein 17-beta-hydroxysteroid dehydrogenase 13 (HSD17B13).

Figure 3A demonstrates the greater than 90% HiFi Assembly efficiency we typically observe when cloning into the F2F vectors, which means just two colonies need to be checked to ensure finding a positive clone. Figure 3B shows the results of our small-scale transient test expression, with MSMS confirming the expression of 15/18 constructs and 9/11 genes. It is to be noted that the expressions of TLR4 (lane 3) and MD-2 (lane 4) can be rescued by switching the His-Twin-Strep tag for a Protein A tag (see Figure 4); therefore, we eventually expressed all 11 of our pilot targets using the F2F methodology.

### 3.4 Representative Stable Cell Lines Yield Homogenous, Active Protein

Following a positive result in the transient test transfection stage, it is necessary to select polyclonal stable cell lines which can be used for large-scale purification. At this stage, constructs can also be co-expressed with a binding partner cloned into pExpreS^2^-2, which is G418^R^ (see Table 1). The F2F method includes no optimization of the protocol for selecting stable cell lines as ExpreS^2^ion’s recommended method is sufficient for our applications and works routinely in our hands. The selection of stable cell lines requires 5 weeks, with the actual hands-on time less than 3 h per week. The resulting polyclonal cell lines can be frozen in DMSO for long-term storage without a pronounced decrease in target expression. Stable cell lines can be harvested and passaged twice a week for up to 6 weeks before the expression levels decrease, facilitating relatively simple and high yielding purification “runs.” Given that the expression levels in ExpreS^2^ are often >10 mg/L and cell supernatants can be harvested twice a week, it is reasonable to expect >50 mg of the purified protein after a 4-week purification run for well-expressing targets.

To demonstrate the utility of purifying in bulk from stable cell lines, we scaled up two of our pilot targets, TLR4 and MD-2. MD-2 is the obligate co-receptor for the innate immune receptor TLR4, and the two proteins are known to assemble into a high-affinity two-way complex at the cell surface, which is responsible for the detection of endotoxin (LPS) (Park et al., 2009). Despite testing >100 constructs, we could not express TLR4 or MD-2 in *E. coli*, Sf9, High-Five™, or Expi293(TM) cells. However, using F2F we could express TLR4 and MD-2, both alone and in complex, using a thrombin-cleavable Protein A tag (Figure 4A). The purified proteins are homogenous and monodisperse after size-exclusion chromatography (Figure 4B) and separately purified TLR4 and MD-2 assemble into a complex as expected (Figure 4C). Furthermore, in a solid-phase ELISA that measures TLR4 binding to a known ligand, the fibrinogen-like globe domain of tenascin C (FBG-C) our S2-derived TLR4 is comparable, if not superior, to commercially available mammalian-derived TLR4 from R&D Systems (Figure 4D).

**FIGURE 5:**
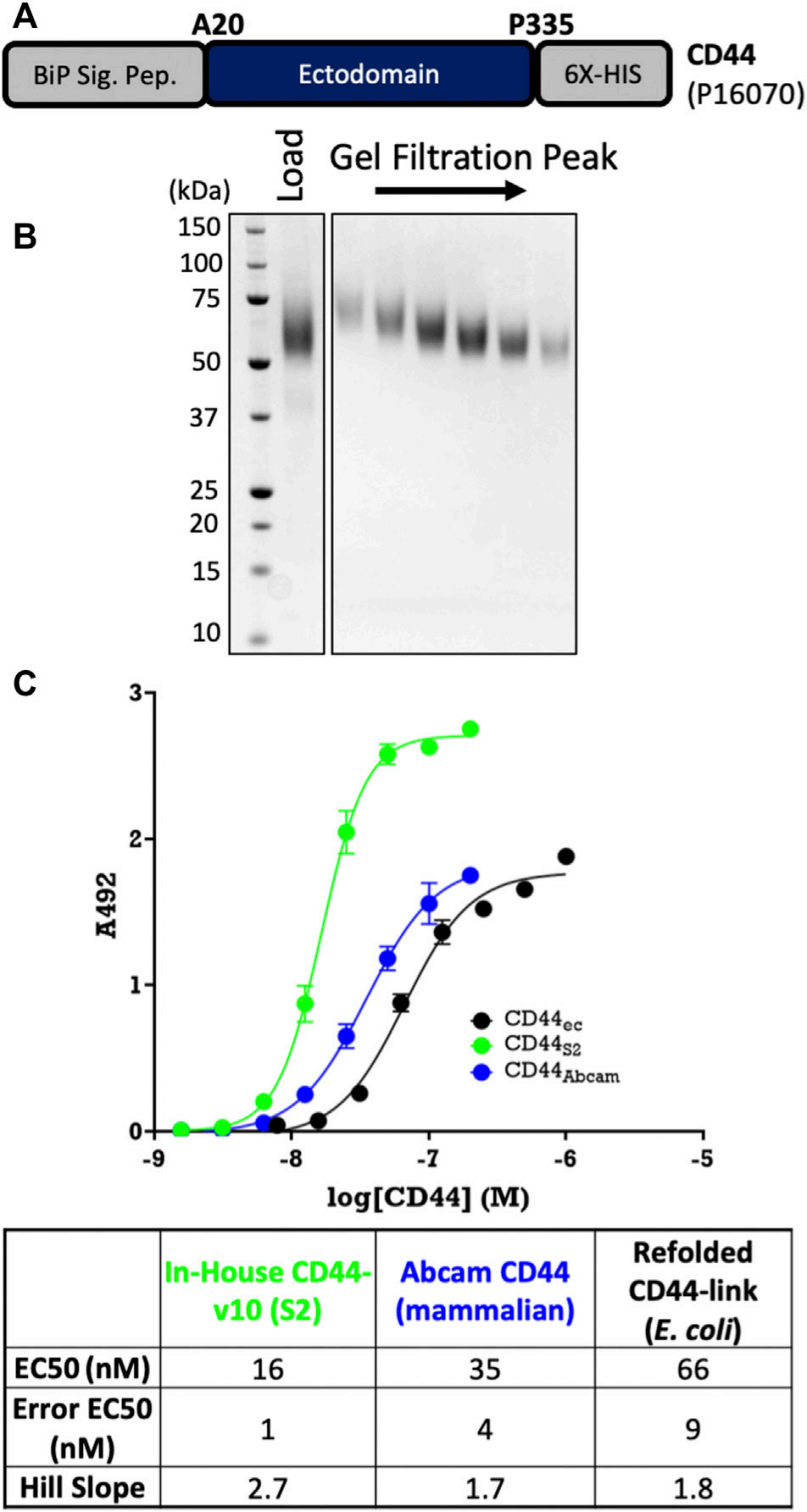
Large-scale purification of CD44. **(A)** Construct design using pExpreS^2^-1-C3-10H-SIII. **(B)** CD44 is pure and monodisperses after SEC. **(C)** Binding assay confirms that S2-derived CD44 binds to its ligand hyaluronic acid (HA) with similar affinity as *E. coli*– or mammalian-derived CD44. Data are presented from a single biological replicate and were fit *via* four component (variable slope) nonlinear regression using GraphPad Prism.

Furthermore, we also scaled up the ectodomain of CD44, which expresses insolubly and with low refolded yields in *E. coli*. Using a non-cleavable 6His tag (Figure 5A), which is standard for this target, we obtained monodisperse proteins at good yields (10 mg/L) following an affinity pull-down on Ni-NTA resin and a gel-filtration clean-up step (Figure 5B). We also showed that S2-derived CD44 binds its ligand, hyaluronic acid (HA), with higher affinity than either *E. coli*–derived re-folded protein or commercially available mammalian-derived protein from Abcam (Figure 5C). Therefore, even for previously intractable targets such as TLR4 and MD-2 and difficult refolded targets such as CD44, stably expressing ExpreS^2^ cell lines yield homogenous, active protein in excellent yields (final purified yield greater than 5 mg/L in all cases).

## 4 Discussion

**FIGURE 6:**
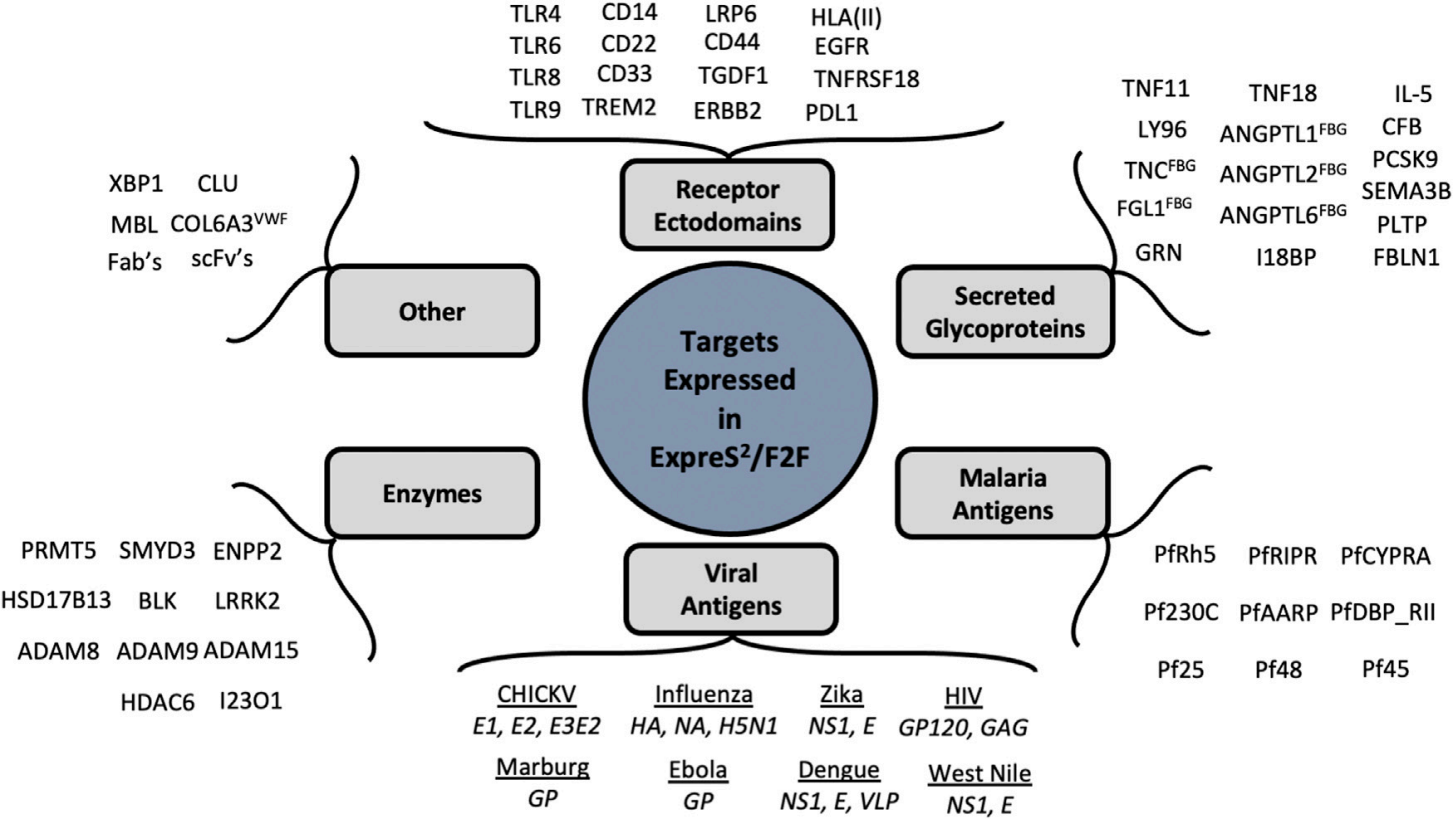
Diverse targets expressed in S2 Cells. Representative examples of proteins expressed with the ExpreS^2^/F2F system both in-house and at ExpreS^2^ion Biotechnologies.

Protein secretion represents a powerful method for expressing challenging targets, particularly disulfide-bonded, posttranslationally modified, or partially disordered proteins as well as receptor ectodomains and naturally secreted proteins. S2 cells, derived from *Drosophila* macrophages, are uniquely tuned protein secretors, and in our hands express ∼90% of “secreted or secretable” targets. Figure 6 provides a sampling of more than 300 proteins that have now been expressed in the ExpreS^2^ system, many of which were intractable in orthogonal eukaryotic expression systems.

The FAS2FURIOUS method represents a step forward versus previously reported S2 systems, with rapid and parallelizable expression testing now feasible for up to 46 constructs in just 2 weeks. We include in Supplementary Material a detailed step-by-step protocol which outlines the entirety of the F2F protocol in granular detail, and all F2F vectors are freely available from Addgene. F2F lays the foundation for the broad implementation of secreted expression pipelines, especially in structural and biophysical laboratories, and should greatly expand the targets amenable to characterization. Given the benefits of F2F in terms of simplicity, yield, speed, and versatility, we expect protein expression in S2 to “catch-up” to more common approaches in Sf9 and High Five™ cells, especially for the most challenging targets.

## Data Availability

The datasets presented in this study can be found in online repositories. The names of the repository/repositories and accession number(s) can be found in the article.
