# Supplementary material for: FAS2FURIOUS: Moderate-Throughput Secreted Expression of Difficult Recombinant Proteins in Drosophila S2 Cells
Source: Front Bioeng Biotechnol. 2022 May 5;10:871933. doi: 10.3389/fbioe.2022.871933 (PMC9117644; doi:10.3389/fbioe.2022.871933)
Supplement: Supplementary file 1 [file DataSheet1.PDF]

FAS2FURIOUS: Moderate-throughput secreted expression of difficult recombinant proteins in *Drosophila* S2 cells

Detailed Supplemental Methods: The FAS2FURIOUS Pipeline

Step 1. Designing and Cloning Constructs

1) Select destination vector from Figure 2A. During primer design, we recommend a 17-25 bp annealing region to your gene of interest. Use the 5'-overhangs recommended in **Table 1,** which when used in a HiFi reaction will result in removal of all cloning scars and ***seamless fusion*** of the insert to an N-terminal signal peptide and C-terminal tags. Alternatively, use NEBuilder to optimize for insert of interest and select "remove restriction scar". (https://nebuilder.neb.com).

Vector	5' Forward Overhang	3' Reverse Overhang
pExpreS2-1-C3-10H-SIII	5'- cctttgttggcctctcgctcggg	5'- ccctgaaacagaacctccaa
pExpreS2-1-C3-PA-10H-SIII		
pExpreS2-1-C3PA		
pExpreS2-1-CRPA		5'- gccgatccgcgtggcaccag
pExpreS2-1-CRPA-10H-SII		

2) ***We recommend handling either 22 (one miniprep cycle, one plate transient transfection) or 46 (two miniprep cycles, two plate transient transfection) constructs at a time.***

3) In a 96-well "primer plate", mix forward and reverse primers for each construct to a final concentration of 10 µM by mixing 10 µL of each primer with 80 µL of nuclease-free water.

a) Set-up a high-fidelity PCR reaction in 96-well PCR plates. We recommend using NEB Q5 High-Fidelity DNA Polymerase. Example recipe:

**Component**	**Volume to Add** (µL)
5X Q5 Reaction Buffer	5
10 mM dNTP Mix	0.5
5X GC Enhancer	5

2.5 ng/µL Template DNA	2.5
10 µM Primer Mix from Primer Plate	1.25
Nuclease-free Water	10.5

4) In a thermal cycler, amplify constructs. We recommend using a modified Touchdown PCR to accommodate differential annealing temperatures between constructs.[29] Example thermal cycling conditions for Q5 High Fidelity DNA Polymerase:

**Initial Denaturation** 1 Cycle	98°C for 10 min
**Stage 1** 5 Cycles	98°C for 30 sec<br>***65°C for 30 sec***<br>72 °C for 1-3 min (30 sec/ kb)
**Stage 2** 5 Cycles	98°C for 30 sec<br>***60°C for 30 sec***<br>72 °C for 1-3 min (30 sec/ kb)
**Stage 3** 5 Cycles	98°C for 30 sec<br>***55°C for 30 sec***<br>72 °C for 1-3 min (30 sec/ kb)
**Stage 4** 15 Cycles	98°C for 30 sec<br>***50°C for 30 sec***<br>72 °C for 1-3 min (30 sec/ kb)
**Final Extension** 1 Cycle	72°C for 10 min

5) After PCR is finished, mix 3 µL of PCR product with 5 µL nuclease-free water and 2 µL of 5X Sample Loading Dye and visualize via agarose gel electrophoresis.

6) Add Dpn1 to PCR products and incubate at 37°C for 1 hour. Store amplified, un-purified PCR products at -20°C

7) Repeat Steps (3)-(5) for any unamplified constructs. We recommend not moving forward with downstream cloning until all PCR products are ready. Potential optimization parameters:

a) Annealing temperature

   b) Exclusion of 5X GC Enhancer, or alternate additives like betaine or DMSO
   c) Doubling the amount of primers and/or template

8) Linearize pExpreS2-1 to prepare for HiFi assembly. We recommend an overnight digestion with BglII at 37 °C to minimize background uncut vector. Example digestion mix:

Component	Amount to Add
10X NEB Buffer 3.1	5 µL
pExpreS2-1-X Vector	0.5 µg
NEB BglII	2.5 µL
Nuclease-free Water	Up to 50 µL

9) Confirm linearization of the vector via agarose gel electrophoresis (3 µL of digestion with 5 µL nuclease-free water and 2 µL of 5X Sample Loading Dye). No uncut (i.e. supercoiled) vector should be visible in the sample.

10) Spin purify the linearized vector using a commercial PCR Clean-up Kit. We recommend eluting the vector in 35 µL of nuclease-free water. Record the concentration of the linearized vector using a NanoDrop.

11) Remove the frozen, un-purified PCR products from the freezer and thaw on benchtop at RT.

12) In a 96-well PCR plate, set up a ½ volume HiFi reaction for each construct using the following recipe:

Component	Amount to Add
2X NEB HiFi Master Mix	5 µL
Linearized Vector	50 ng
Un-purified PCR Product	1 µL for bright bands<br>2 µL for faint bands<br>(*maximum amount of un-purified PCR product is 2 µL)*
Nuclease-Free Water	Up to 10 µL

13) Incubate the HiFi reaction in a thermal cycler for 30 minutes at 50°C to allow annealing and ligation of the inserts into the linearized vector. For more details on the HiFi reaction, see:

https://www.neb.com/applications/cloning-and-synthetic-biology/dna-assembly-and-cloning/nebuilder-hifi-dna-assembly

14) While the HiFi reaction proceeds, thaw any commercially available chemically competent cloning cell line (Mach 1, DH5α, TOP10, ect.) on ice. We use homemade Mach 1 cells. Highly competent cells are paramount at this step.

15) In a 96-well PCR plate, aliquot 40 µL competent cells into each well, one for each HiFi reaction and two additional wells for negative controls.

16) Using a multichannel pipette, add 2 µL of the finished HiFi reaction to the competent cells. Add 1 µL of the linearized vector to one well and leave one well as cells only for negative controls.

17) The unused HiFi reaction and linearized vector can be stored indefinitely at -20°C.

18) Incubate the cell/DNA mixture on ice for 30 min before heat shocking for 45 seconds in a 42°C water bath. Return to ice for 2 minutes.

19) Add 100 µL of SOC or 2X LB to each well and then allow the cells to recover for 1 hour at 37°C.

20) Plate 75 µL of the transformation mixture onto pre-prepared low-salt, 1.5% LB-Agar plates containing 25-50 µg/mL Zeocin. Note that Zeocin is heat sensitive, so ensure LB-agar mixture is cooled to 55°C in a water bath before adding the Zeocin. Zeocin plates can be stored in the dark at 4°C for up to two weeks. Recipe for low-salt LB-Agar:

**Component**	**Amount to Add**
Tryptone	10 g/L
NaCl	5 g/L
Yeast Extract	5 g/L
pH	To 7.5

21) Incubate plates overnight at 37°C. Check for colonies in the morning.

a) We often observe a few background colonies (< 15 and clearly fewer than on the experimental transformation plates) in the linearized vector negative control plate. This stems from trace amounts of uncut vector and is normal. Insert-containing clones will be selected in Step II.

22) Seal plates with parafilm and store at 4°C for up to two weeks. Repeat transformation with 4 µL of the HiFi reaction for any plates without colonies. Alternatively, HiFi reaction can be optimized around the following parameters:

a) Extend HiFi reaction time to 1 hour

This is a provisional file, not the final typeset article

b) Spin purify the PCR products and adjust the ratio of insert:vector (NEB recommends 2 insert: 1 vector molar ratio) based on insert length using actual concentrations

Step 2. Checking for Inserts and Miniprepping Constructs

1) Once colonies are obtained on all plates, proceed with preparation of the constructs for transfection. Remove plates from 4°C and allow to equilibrate to RT.

2) Prepare 10 μM primer master mix by mixing 20 μL of each primer with 160 μL nuclease-free water. Use the colony PCR primers (pExpreS2-1-for1 and pExpreS2-rev1) listed below, which when used together add 240 bp to the theoretical construct length.

**Primer Name**	**Sequence**	**Location**
pExpreS2-1-for1	5'-acaagacaggtttaaggagac	Upstream synthetic intron
pExpreS2-2-for1	5'-ccggagtataaatagaggcgc	Upstream HSP70 promoter
pExpreS2-rev1	5'-gcgcttgaaaggagtgtgta	Downstream opie pAA signal

3) Prepare a colony PCR master mix according to the manufacturer's instructions. We recommend Bioline My Taq Red Colony PCR Kit. Example Recipe:

**Component**	**Amount to Add** (μL/rxn)	**Amount to Add** (μL/100 rxn)
Bioline 5X Buffer	4.0	400.0
Nuclease Free Water	14.9	1,490.0
10 μM Primer Master Mix	1.0	100.0
Bioline MyTaq DNA Polymerase	0.1	10.0

4) Aliquot 20 μL of the Colony PCR Master Mix prepared in step (3) into a 96-well PCR plate. We recommend preparing 2 wells for each cloned construct as well as one control well for each vector used. For simplicity, ***this means cloning no more than 46 constructs at once.***

5) Prepare an outgrowth plate using a 2 mL deep-well 96-well block. Add 1 mL of 1X LB + 25-50 µg/mL Zeocin to each well.

6) Using a fresh 96-tip rack of 200 µL tips, pick two colonies from each construct plate. First, dip the tip into the PCR Master Mix and swirl five times. Then, place the tip in the corresponding well in the deep-well outgrowth plate. We recommend leaving the tips in the deep-well outgrowth plate until all colonies are picked. For the controls, simply add 1 µL of a 2.5 ng/µL stock of the destination vector(s) to the PCR Plate. Example 96-well set-up:

	**1**	**2**	**3**	**4**	**5**	**6**	**7**	**8**	**9**	**10**	**11**	**12**
**A**	1	2	3	4	5	6	7	8	9	10	11	12
**B**	1	2	3	4	5	6	7	8	9	10	11	12
**C**	13	14	15	16	17	18	19	20	21	22	23	24
**D**	13	14	15	16	17	18	19	20	21	22	23	24
**E**	25	26	27	28	29	30	31	32	33	34	35	36
**F**	25	26	27	28	29	30	31	32	33	34	35	36
**G**	37	38	39	40	41	42	43	44	45	46	Control1	
**H**	37	38	39	40	41	42	43	44	45	46	Control2	

7) Remove tips from the deep-well outgrowth plate and place into an incubator/shaker at 37°C. If using a designated plate shaker, we recommend 450 RPM. If using a standard bench-top incubator/shaker, we recommend 200 RPM.

8) Place the PCR Plate in a thermal cycler and amplify the constructs according to manufacturer's instructions. Using Bioling MyTaq:

**Initial Denaturation**<br>1 Cycle	94°C for 10 min
**Stage 1**<br>25 Cycles	94°C for 30 sec<br>50°C for 30 sec<br>72 °C for 1-3 min (30 sec/ kb)
**Final Extension**<br>1 Cycle	72°C for 10 min

This is a provisional file, not the final typeset article

9) Visualize the PCR products by agarose gel electrophoresis by loading 10 µL of the PCR reaction. Compare constructs to theoretical length, remembering that the above colony PCR primers will add 240 bp. The vector-only controls can be used to eliminate any colonies which contain empty (i.e. insert-free) vectors. Typically, we observe < 10% background, meaning that selecting 2 colonies/construct is sufficient.

10) Repeat Steps (3) – (8) for any constructs which did not yield a positive clone. We recommend picking 3-6 colonies for any constructs which are negative in the first colony PCR.

11) Prepare overnight culture block(s) using 24-well deep well plates. Aliquot 4 mL of 1X LB + 25-50 µg/mL Zeocin into each well (one well per construct). **Carefully** move the 1 mL outgrowth culture from positive colonies into the 4 mL overnight culture block. It is critical to move only the positive clones at this stage!

12) Grow the 5 mL overnight cultures in an incubator/shaker at 37°C. If using a designated plate shaker, we recommend 450 RPM. If using a standard bench-top incubator/shaker, we recommend 200 RPM.

13) The next morning, mini-prep the constructs using spin columns according to the manufacturer's instructions. Note that filter-plate-based miniprep kits *do not* yield enough DNA for storing and transfection. We recommend two cycles, wherein 2 X 23 constructs are mini-prepped simultaneously. We recommend eluting in 35 µL TE to maximize concentrations.

14) Check and record the concentration of all the constructs. On average, we observe 250-500 ng/µL of DNA for pExpreS2-1 backbone vectors (they are very high copy number). *Note that any constructs < 200 ng/µL may lead to false negatives in the test expression stage due to insufficient DNA. We recommend moving forward even with low yield, and considering re-prepping the construct only if the test expression is unexpectedly negative.*

15) Move the eluted DNA into a fresh 96-well PCR plate.

16) In a fresh, clean, v-bottom 96 well plate, aliquot 21.5 µL of TE (one well per construct). Using a multi-channel pipette, move 3.5 µL of the miniprep DNA into the v-bottom storage plate. This plate can be stored at -20°C indefinitely.

17) Heat-sterilize the remaining miniprep DNA in the 96-well PCR plate by incubating at 95 °C for 10-15 min. This sterilized DNA will be used in Step 3 for the transient transfection. DNA can be stored at -20°C indefinitely but should not be opened outside of a cell culture hood from this point forward.

Step 3. Transient Transfection in 24-well Plates (TC Hood Required)

1) Split S2 maintenance cell lines to 8 X 10^6 cells/mL in Excell420 + Pen/Strep. Prepare 75 mL of cells per 24-well plate to be used.
2) Into each well of a sterile, deep-well, round-bottom, 24-well plate, add 3 mL of diluted S2 cells. For 22 constructs, use 1 block, for 46 constructs, use 2 blocks.
3) Add 37.5 µL of ExpreS^{2}ion 5X Transfection Reagent (5X TR) to each well by carefully dripping from above. Vigorously mix the plate (>50 times), careful not to spill any of the cells out of the plate.
   a) Note that alternative transfection reagents are also amenable to this protocol (we have successfully used JetPrime in house). However, this protocol has been optimized for 5X TR. We recommend experimentally optimizing the cells:DNA:transfection reagent ratio if an alternative transfection reagent is substituted at this stage.
4) From the heat-inactivated plasmid DNA miniprep plate, add 7.5 µg DNA of each construct to the corresponding well. For any constructs with < 7.5 µg total DNA, transfect everything and understand that false negatives are a possibility. We recommend gently shaking the plate 3-5 times after each pipetting step to mix the DNA/TR.
   a) There are two choices at this stage. Either add DNA individually to each well, after calculating the exact volume required for 7.5 µg. Alternatively, average the DNA concentrations across the plate and add an equal volume to each well using a multi-channel pipette. We recommend standardizing the 7.5 µg for robust, comparative test expressions; however, this approach does take significantly more time.
5) Prepare two control transfections by adding (1) just transfection reagent to one well and (2) 7.5 µg of a control construct with known expression levels (we use TLR4 in-house). You can also include a transfection control at this stage by adding (3) a pExpreS2-1-GFP control. However, even with low transfection efficiencies it we recommend proceeding with the test purification, so we often just perform controls (1) and (2).
6) After all the DNA is added, rest the plate(s) at RT for 5 minutes.
7) Cover the plate(s) with a sterile air-porous seal and then incubate in an incubator/shaker at 25°C and 500 RPM. Rapid stirring is essential at this stage to ensure the cells remain in suspension; we

This is a provisional file, not the final typeset article

strongly recommend a designated plate-compatible incubator/shaker for this step. Allow the cells to grow for 3 day (68-72 hours).

8) After 3 days, observe the cells under a microscope to check for contamination. Also record any wells with an abundance of dead cells, suggesting a toxic insert. If no contamination is observed, proceed with Step 4.

Step 4. Test Purification Using Deep-Well Filter Plates

1) Pellet the cells by spinning at 1500 x *g* for 20 min at RT. Ensure that a plate-compatible centrifuge is used.

2) In a separate 24-well deep well block(s), add 100 μL bed volume of purification resin pre-equilibrated in 1X PBS (or equivalent, like HBS) into each well.

a) Any resin works at this stage. We recommend StrepTactin®XT Fast Flow resin for twin-strep tags, Ni-NTA or TALON™ resin for His-tagged protein, or IgG Resin for Protein A tags. StrepTactin®XT and IgG pull-downs are much cleaner, and we recommend using these tags for large purification. However, the Ni-NTA purifications are sufficient for test purifications, even in cell culture media, because of the strength of the 10His tag.

b) *Note: if performing a StrepTactin®XT pull-down, be sure to neutralize the free biotin in Excell420 by adding BioLock according to manufacturer's recommendations. Excess free avidin protein also blocks the biotin well. Otherwise, the free biotin from the cell culture media will prevent binding to resin.*

3) Using a multi-channel pipette, prepare a "total secretome" sample by mixing 5 μL of supernatant with 10 μL 1X PBS and 5 μL 4X Laemmli Loading Dye.

a) Note that we often do not see any target protein in these samples because of the large amount of background secreted proteins.

4) Using a multi-channel pipette, gently transfer the supernatants into the 24-well block(s) with pre-equilibrated resin. Do not disturb the pellet, as chunks of cells can block the filter plate.

5) Place the resin/supernatant mixture at 4°C and shake at gently for 1 hour to allow binding.

6) Place a 96-well deep well filter plate (such as ThermoFisher 278011) over a second 96-well deep well waste plate.

7) Gently pellet the bound resin by spinning at 700 x *g* at 4°C for 5 minutes.

8) Gently remove the supernatant (flow-through) without disturbing the pellet. It is fine not to remove every bit of the supernatant to avoid disturbing the resin.

9) Add 750 µL of 1X PBS to the 24-well blocks and re-suspend the resin. Transfer bound resin into the 96-well deep well filter plate. *Note that transferring from a 24 to 96-well plate results in adjacent wells from the 24 well block becoming "every-other" in the 96-well plate. See diagram below for clarification.*

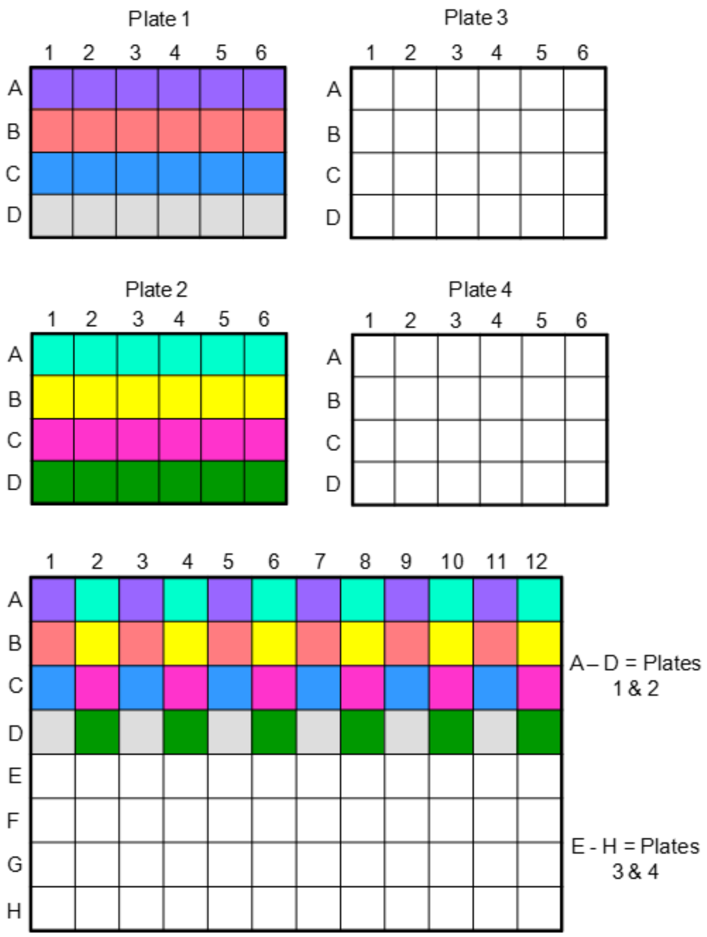

10) Allow the buffer to drip through or centrifuge at 200 x *g* for 2 minutes.

11) Discard flow-through. Wash the resin with 800 µL 1X PBS. Allow the buffer to drip through or centrifuge at 200 x *g* for 2 minutes.

12) Wash the resin as before with 4-5 X 800 µL Wash Buffer. For IgG or StrepTactinXT pull-downs, the wash buffer is simply 1X PBS. For NiNTA pull-downs, we recommend 30 mM imidazole. For TALON pull-downs, we recommend 10 mM imidazole.

13) After washing, place the filter plate on top of a 96-well v-bottom microtitre plate. Add 100 µL (1CV) of corresponding elution buffer to each well of the filter plate. Incubate with vigorous shaking at RT for 15 minutes. Recommended elution buffers:

a) StrepTactinXT: 50 mM D-Biotin in 1X PBS

   b) NiNTA: 500 mM imidazole in 1X PBS
   c) TALON: 250 mM imidazole in 1X PBS
   d) IgG: 0.5 M Acetic Acid, pH = 3.4

14) Collect eluted protein inot the 96-well microtitre plate by centrifuging at 500 x *g* for 5 minutes.

15) Prepare SDS-PAGE samples by mixing 15 µL of elution with 5 µL 4X Laemmli loading buffer.
   a) At this stage, you can also prepare a "deglcosylated" sample by mixing 14 µL with 1 µL of ~ 1 mg/mL PNGaseF and 5 µL 4X Laemmli loading buffer. We purify PNGaseF in-house and see no loss of activity even in the loading buffer.

16) Analyse the eluted protein on SDS-PAGE. Compare to the background negative-control pull-down; however, ***we strongly recommend validation of putative target bands by in-gel digest MSMS***, particularly for weak expressers.

Step 5. Stable Cell Selection

Note: This protocol is almost identical to the standard protocol included with the ExpreS^{2}ion Biotechnologies Platform. It is re-produced here for simplicity.

1) This step should be completed for well-expressing constructs needed for scale-up. We recommend selecting no more than 4 stable cell lines at once, especially the first time through the protocol.

2) Constructs of interest should be re-transformed and Midi-Prepped or Maxi-Prepped according to the manufacturer's instructions. Plasmid DNA should preferably be between 0.6—1.0 µg/µL and endotoxin free.

3) **Day 0**. Split S2 cells by centrifugation (450 x *g* for 3 min) to a final density of $5x10^6$ per mL in Excell420 + 10% FBS in a shake flask. Usually a 50 mL culture is more than sufficient.

4) **Day 1.** Split the cells be centrifugation as above, down to a final density of $2x10^6$ per mL in Excell420 *without FBS*.

5) Transfer 5 mL of $2x10^6$ S2 cells to a T25 T-flask, one flask per transfection. Also include (1) a positive control flask to be transfected with pExpreS2-1-GFP and (2) a negative control flask to be mock transfected.

6) For each flask in succession, add 50 µL of ExpreS^{2}ion 5X-TR and gently swirl the T-25. Add 12.5 µg of DNA to flask and swirl again. Rest 5 minutes, then incubate without shaking at 25°C for 4 hours.

7) After 4 hours, add 1 mL of FBS.

8) **Day 2.** Check the positive control flask under a fluorescent microscope to get an idea of transfection efficiency. We routinely see >50% GFP-positive cells, although even ~10% is sufficient to select a stable cell line.

9) Add the selection reagent. For pExpreS2-1 class vectors, use 2 mg/mL Zeocin. For pExpreS2-2 class vectors, use 4 mg/mL G418. For co-selection, use 1.5 mg/mL Zeocin + 4 mg/mL G418.

10) **Day 4-24.** Count the cells every 3-4 days (we check on Mondays and Fridays). If the cell density is > $1x10^6$, dilute the cell suspension by removing cells and adding fresh Excell420 + 10% FBS + selection marker. Split the negative control flask in the same manner as the experimental flasks.
    a) Especially after the first few days, the split is often large (i.e 1 mL cell suspension + 5 mL fresh media). It is important to keep the cells dilute to allow effective selection.

11) **Day 24.** At this point, the negative control flask should be completely dead and 100% of the positive control flask should be GFP-positive under a fluorescent microscope.
    a) After low efficiency transfections, we sometimes extend to a 28-day selection.

12) Transfer the 6 mL culture from a T25 into a T75 and add 9 mL of Excell420 + 10% FBS.

13) **Day 26 and on**. Expand the cells to 25 mL volume in 125 mL shake flask + 10% FBS. After one passage, remove the FBS. We recommend freezing the cells at this point using standard methods (like 10% DMSO), before scaling up to larger volumes for purification.

14) At this time, you can take a sample of the supernatant and carry out a test pull-down as in Step 4 to ensure expression. ***We have never observed a positive construct from the small-scale transient test that did not express similarly as a stable cell line.***

Step 6. Large-Scale Expression Purification

1) Stable cell lines can be cultured up to 1L volumes in 3L flat-bottom shake flasks. We routinely passage stable cell lines to $5x10^6$ cells/mL by centrifugation (450 x *g* for 3-5 min) every Monday and Friday, harvesting the supernatant for purification. Follow these guidelines when deciding which size flask to use:

Flask Size	Optimal Vol. (mL)	Min Vol. (mL)	Max Vol. (mL)	Optimal rpm
RK125	25	20	30	115
RK250	50	30	60	115

This is a provisional file, not the final typeset article

RK500	100	60	150	115
RK1000	200	150	300	115
RK2000	400	300	550.00	130
RK3000	1000	500	1200	130

2) Protein can be purified as normal using standard approaches. We often perform a single-step pull down on either IgG or StrepTactin®XT resin followed by tag cleavage and size-exclusion chromatography.

- **a)** As mentioned in Step 4-2, be sure to neutralize the free biotin in the cell culture media before pulling down protein on StrepTactin®XT resin!
- **b)** We recommend filtering the supernatant before purification to remove any cellular debris.
- **c)** Because of the large volume of media to be purified, we do not recommend harvesting more than 2 L of cells from shake flasks at a time.
- **d)** Batch-fed and fermentation systems also work with the stable cell lines; however, we have never tried these methods in-house.

Stable cell lines can be continually passaged and used for purification for up to four consecutive weeks in our hands. After that point, expression levels begin to decline, at which point we thaw a new vial from the freezer.
